# *PolyAdapt*: Characterizing Polygenic Adaptive Architectures in the Presence of Strong Linkage Disequilibrium

**DOI:** 10.1093/gbe/evag132

**Published:** 2026-06-09

**Authors:** Rupert Mazzucco, Christian Schlötterer

**Affiliations:** Institut für Populationsgenetik, Vetmeduni Vienna, Wien 1210, Austria; Institut für Populationsgenetik, Vetmeduni Vienna, Wien 1210, Austria

**Keywords:** polygenic adaptation, 2 genotypes, experimental evolution, haploblocks, simulation

## Abstract

Characterizing the genetic architecture of adaptation remains a central challenge in population genetics, particularly when multiple loci contribute to selected phenotypes. Two-genotype experimental evolution studies offer a powerful framework for this purpose, yet existing methods lack the capacity for genome-wide inference of selection targets and their coefficients under linkage. Here, we introduce *polyAdapt*, a novel iterative algorithm that identifies selected haplotype blocks and estimates the associated selection coefficients from Pool-Seq data in two-genotype experiments. Rather than exploring all possible combinations of selection targets simultaneously, *polyAdapt* sequentially incorporates targets of decreasing effect, accounting for linked selection at each step and optimizing both selection coefficients and effective population size through comparison of empirical and simulated replicate allele frequency trajectories. Using simulated data sets, we demonstrate that *polyAdapt* accurately recovers selection targets and coefficients for oligogenic architectures (5 and 13 targets) and provides a lower bound on the number of contributing loci for polygenic architectures (50 targets). Applied to yeast experimental evolution data, *polyAdapt* infers a highly polygenic architecture with at least 20 selected haplotype blocks per chromosome, consistent with independent estimates from the literature. *polyAdapt* thus represents a flexible and powerful tool for dissecting the genetic architecture of adaptation in experimental evolution studies.

Significance Understanding how organisms adapt at the genetic level is challenging when many genes contribute simultaneously, because their effects are difficult to disentangle from random genetic drift. We developed *polyAdapt*, a computational method that identifies genomic regions under selection and estimates their strength by iteratively analyzing allele frequency changes in controlled evolution experiments which started with just two genotypes. Applied to simulated and real yeast data, *polyAdapt* reliably recovers the targets and strength of selection for simple architectures and establishes minimum bounds on complexity for highly polygenic adaptation, providing new insight into the genomic basis of evolutionary change.

## Introduction

Molecular population genetics has a long tradition of identifying selection signatures from polymorphism data (Thornton et al. 2007; Casillas and Barbadilla 2017). Because natural populations often have very large sizes, linkage is usually disregarded in many neutrality tests, and each single-nucleotide polymorphism (SNP) is examined independently of the others (Pavlidis and Alachiotis 2017). However, some population genetic tests explicitly take advantage of linkage disequilibrium to distinguish selection from neutral scenarios (Voight et al. 2006; Sabeti et al. 2007). But even these tests do not account for linkage among multiple selected loci. A major breakthrough was the introduction of the ancestral recombination graph and the development of inference approaches (Rasmussen et al. 2014; Kelleher et al. 2019; Speidel et al. 2021; Martin 2026). Hence, the focus has shifted from the analysis of presumably independent SNPs to the inference of selection signatures of haplotype blocks. The important distinction is that the combined effect of all selection targets in a haplotype block is considered rather than attempting to separate them. Since this field of research is still in its infancy, it is currently unknown whether the reconstruction of the ancestral recombination graph is sufficiently accurate to build reliable models that jointly model demography and selection and provide reliable selection estimates.

Small populations face two compounding challenges: strong genetic drift and few recombination events. Most experimental evolution studies of sexual organisms operate under precisely these conditions, with typical population sizes of around 1,000 interbreeding individuals (Burke et al. 2010; Orozco-terWengel et al. 2012; Griffin et al. 2017; Michalak et al. 2019). One common strategy to compensate for limited within-experiment recombination is to exploit the diversity accumulated in natural populations by starting experiments with highly polymorphic populations derived from a large number of isofemale lines (Kofler and Schlötterer 2014). Although this approach has proven effective for traits with a simple genetic basis (Martins et al. 2014), it does not generally resolve the recombination problem. More commonly, rare haplotypes rise in frequency during experimental evolution, producing large selected haplotype blocks spanning multiple megabases—a direct consequence of the limited recombination events occurring during the spread of a beneficial allele (Franssen et al. 2017a; Barghi et al. 2019).

Polygenic adaptation in experimental evolution studies with multiple founder genotypes introduces a further layer of complexity. Unlike biallelic selection targets, haplotype blocks may carry different combinations of advantageous and disadvantageous alleles, such that multiple blocks of varying fitness compete against one another, analogous to a multiallelic locus. Interpreting selection signatures under these conditions is further complicated by the widespread use of Pool-Seq (Schlötterer et al. 2014), an approach in which pools of individuals are sequenced together with short reads. Although Pool-Seq delivers reliable genome-wide allele frequency estimates, analyzing the resulting data at the level of individual SNPs rather than haplotypes gives rise to complex and difficult-to-interpret patterns of allele frequency change (Barghi and Schlötterer 2019).

Many of the problems associated with experimental evolution involving multiple founder genotypes can be overcome by using only two distinct haplotypes. Clegg and colleagues proposed this radically simple founder population structure and explored it using allozyme markers and computer simulations (Clegg et al. 1976, 1979, 1980; Cavener and Clegg 1978; Clegg 1978). More recently it has been shown that genome-wide allele frequency trajectories can easily be followed with Pool-Seq (Kosheleva and Desai 2018; Burny et al. 2021, 2022).

Two-genotype experiments combined with Pool-Seq offer three key advantages over experimental evolution studies with complex founder populations. First, allele frequencies can be estimated with greater accuracy, because the strong correlation among neighboring SNPs allows averaging across multiple loci. Second, having only two founder genotypes substantially simplifies the modelling of recombination and selection. Third, an equal starting frequency of 0.5 for both genotypes reduces heterogeneity among replicates caused by genetic drift (although the sampling variance is higher), resulting in more parallel selection responses across replicates (Burny et al. 2021).

The conceptual advantages and challenges of characterizing adaptive architectures with two-genotype experiments are most clearly understood by considering architectures of increasing complexity: simple genetic architectures, in which no more than one selection target is present per chromosome; oligogenic architectures, characterized by a small number of selection targets; and highly polygenic architectures, in which many loci of individually small effect contribute to the selected phenotype.

Simple adaptive architectures harboring a single selection target per chromosome are the most straightforward to characterize: the favored allele is expected to show the most pronounced frequency increase, and the distinction between the selection target and hitchhiking neutral alleles improves with larger population sizes and longer experiments. Selection responses driven by such simple architectures can therefore be reliably studied with standard approaches (Vlachos et al. 2019).

Oligogenic architectures present a greater challenge. When multiple selection targets reside on the same chromosome, they are not independent of one another due to the limited recombination inherent to two-genotype experiments. As a result, the frequency change observed at any given locus reflects a mixture of direct and indirect selection forces acting on linked targets. Given sufficient generations, recombination progressively uncouples these targets, enabling the characterization of loci separated by enough recombination events. It is evident that the non-independence of selection targets requires an analytical approach that accounts for the confounding effects of linked selection to identify selection targets and to infer their selection strengths.

Highly polygenic architectures pose the greatest challenge to genetic analysis. When many loci of individually small effect contribute to a selected phenotype, the resulting genomic signatures of adaptation are notoriously difficult to distinguish from the effects of genetic drift, even in large populations (Pritchard et al. 2010; Hayward and Sella 2022). Two-genotype experiments offer a distinctive advantage in this context: because only two configurations of the polygenic architecture are present, the selection response at any given genomic region reflects the combined effect of all contributing loci within those two genotypes. Haplotype blocks in which the two genotypes differ substantially in their combined fitness effects will exhibit a strong selection response, even if each individual locus has only a minor effect. Linkage disequilibrium can therefore amplify the genomic signal of polygenic adaptation considerably. This principle is supported by computer simulations demonstrating that clustered loci with aligned fitness effects can drive the introgression of an entire haplotype block, even when the immigrant and recipient populations have identical overall fitness (Sachdeva and Barton 2018).

Two-genotype experiments are thus well suited to characterizing polygenic architectures through genome-wide allele frequency changes, which reflect the distribution of contributing loci across the two founder genotypes. The resolution achievable depends on both the duration of the experiment, which determines how effectively recombination uncouples linked sites, and the configuration of contributing loci on the two founder haplotypes. Critically, selection responses can only be resolved at the level of haplotype blocks rather than individual loci. This means that, at a given generation, it remains impossible to determine whether the observed response is driven by a single locus of moderate effect or by multiple linked loci with an equivalent combined effect.

A range of methods have been proposed to study selected haplotypes in sexual populations using Pool-Seq data, either with (Long et al. 2011; Kessner et al. 2013; Burke et al. 2014) or without (Franssen et al. 2017a; Otte and Schlötterer 2021; Pelizzola et al. 2021; Wang et al. 2024) prior knowledge of the founder genotypes. Moreover, approaches have been suggested that allow the estimation of selection coefficients and take into account that more than one haplotype block can be selected, each with a specific selection coefficient (Chen et al. 2023). Despite their undoubted merit, these approaches suffer from several limitations regarding genome-wide characterization of the genetic architecture. Reconstructing haplotype blocks from Pool-Seq data alone does not identify which blocks are targets of selection or explain the observed allele frequency changes. The method of Chen et al. (2023) is restricted to short haplotype blocks, limiting its ability to characterize the genome-wide architecture of adaptation.

Here, we introduce an entirely new framework for characterizing the architecture of polygenic adaptation, built on the strengths of two-genotype experiments. By leveraging highly accurate genome-wide allele frequency estimates, we jointly infer the number of selection targets and their associated selection coefficients. To avoid the computational burden of exploring a high-dimensional parameter space with an unknown number of targets, we use an iterative strategy that progressively accounts for selection targets in order of their impact on the observed allele frequency trajectories. We demonstrate that this approach yields reliable estimates for oligogenic architectures of varying complexity (5 and 13 selection targets per chromosome). For more complex architectures, only a lower bound for the number of selection targets can be determined; nevertheless, we demonstrate the utility of this approach for even rather complex adaptive architectures and apply it to empirical data from yeast.

## Results

Two-genotype experiments aim to characterize the favored haplotype blocks. Starting with a mass cross of the two homozygous genotypes, the descendants of this cross are propagated in replicate populations that are exposed to the same novel environment across multiple generations. Without recombination, the frequency of an entire chromosome would only change if the combined effect of all selected alleles differs between homologous chromosomes. In the presence of recombination, favored alleles are shuffled onto the same chromosome. This does not only allow for a much stronger and faster adaptive response than in the non-recombining case but also selection targets can be characterized based on the patterns of allele frequency change. When favored alleles alternate between both homologous chromosomes, the genomic selection response resembles a zigzag pattern if the frequency of one of the founder genotypes is plotted across the entire chromosome. However, since recombination is limiting in these experiments, haplotype blocks (ie a junk of chromosome originating from one of the founder genotypes) with multiple alleles are responding to selection. Sequencing pools of individuals (Pool-Seq; Schlötterer et al. 2014) provides the required genome-wide allele frequency data. We developed *polyAdapt*, a novel approach to identifying the haplotype blocks, which contain selection targets and their associated selection coefficients (the combined effect of all contributing loci in the haplotype block) in two-genotype experiments.

### The Algorithm

For computational feasibility, *polyAdapt* does not identify all selection targets in a single step. Rather, the selection targets are detected in an iterative process.

**Fig. 1.**
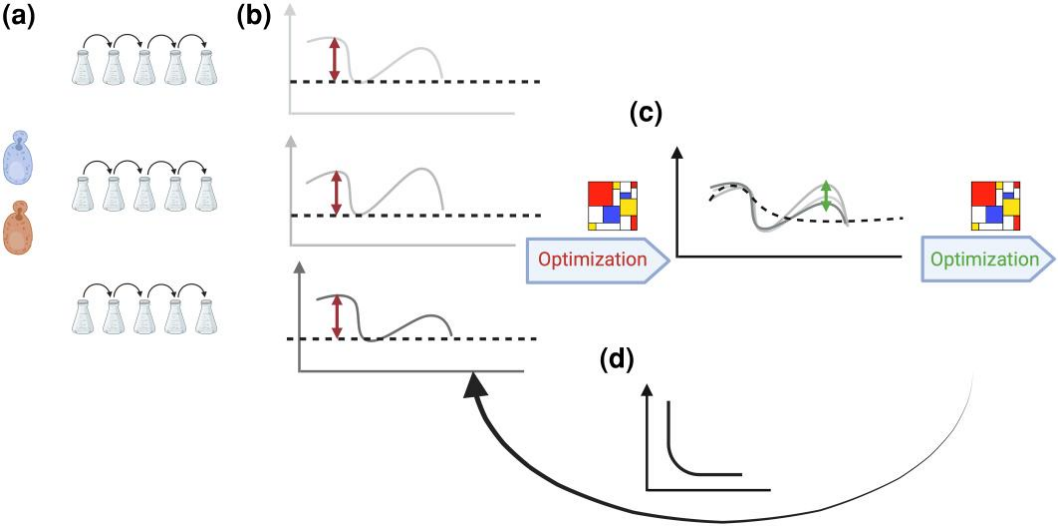
Schematic overview of the *polyAdapt* algorithm. a) Starting from two homozygous genotypes, experimental evolution is performed in replicate populations over multiple sexual generations. Allele frequencies are obtained by averaging the frequency of neighboring SNPs as determined by Pool-Seq. These data are used by *polyAdapt* to infer the number of selection targets, their selection strength, and the effective population size. b) The frequency of one of the founder genotypes along an entire chromosome is shown for three replicates. *polyAdapt* identifies the position in the genome where the empirical frequencies deviate most from the simulations (broken line). The selection coefficient is determined by minimizing the distance between empirical and simulated data at the selection target (red arrow). Simulations are performed with SLiM. c) After optimizing the selection coefficient, the effective population size is determined by minimizing the difference in genome wide variance in allele frequencies across replicate populations between empirical and simulated data (green arrow). d) *polyAdapt* performs multiple rounds and each round one additional target is included. Each round, the optimization is performed on all selection targets. With an increasing number of selection targets, the distance between empirical and simulated results decreases, as indicated by the inset where the distance is plotted against the number of selection targets. Created in BioRender. Schlötterer, C. (2026) https://BioRender.com/zxj1oug.

In the first step, a selection target is identified at the chromosomal position with the largest allele frequency mismatch between empirical and simulated replicates. The associated selection coefficient is then estimated through optimization starting from the assumption of neutrality, such that this mismatch is minimized at the selection target (Fig. 1), accounting for the median frequency differences between empirical and simulated replicates as well as the variances among replicates (see Materials and Methods for more details).

In a second step the effective population size (*N*_e_) is estimated, again through optimization starting from a user-provided estimate, so that also the mismatch between chromosomal average variances in allele frequencies across replicates of empirical replicates and simulated replicates is minimized (Fig. 1). This estimation procedure takes advantage of the relationship between effective population size and genetic drift. Smaller populations will result in larger differences among replicates for the genome-wide allele frequency trajectories. By combining the effect of selection and drift, matching the variance estimate provides a reliable approach to estimating *N*_e_ even for populations with high levels of linkage disequilibrium.

Then, a new cycle of consecutive optimizations for the selection coefficients and *N*_e_ begins. This cycle starts by incorporating an additional selection target at the genomic position showing the largest mismatch between the empirical data and new simulations based on the selection targets and *N*_e_ estimated in the previous cycle. Then the selection coefficient and effective population size are optimized as described above.

Finally, the procedure ends when the user-defined upper limit of selection targets is reached. An approximate estimate for the number of contributing loci is obtained from a range of summary statistics. We explored the functionality of *polyAdapt* using a combination of simulated and empirical data from *Saccharomyces cerevisiae*.

It is clear that with an increasing number of selection targets, the mapping of selection targets becomes increasingly difficult. For a polygenic architecture in particular, multiple selection targets can be co-located on the same haplotype block (when too few recombination events have occurred to separate them). This implies that the position and number of selection targets on this haplotype cannot be resolved.

Hence, given the limited number of recombination events, *polyAdapt* can only estimate a lower bound for the number of selection targets. Furthermore, the position in a haplotype block cannot be faithfully determined, as the unit of inference is the haplotype block and not a single SNP, despite *polyAdapt* using SNPs in the block for the simulations.

### *polyAdapt* on Simulated Data With Known Selection Targets

Our empirical data were obtained from a study that crossed two yeast strains (Kosheleva and Desai 2018). Therefore, we chose simulation parameters that produced selection responses similar in magnitude to those observed in the yeast experiment (note that this only roughly applies to the oligogenic architectures, as the genomic response of the yeast data was highly polygenic; Kosheleva and Desai 2018). Because the alteration of sexual and asexual growth phases in yeast experiments with recombination is difficult to simulate, we restricted our simulations to sexual reproduction cycles. For this reason, the inferred selection coefficients should be interpreted as realized selection coefficients between sexual generations. We consider this a realistic approximation because the population sizes during asexual growth were extremely large, making the influence of genetic drift negligible. The functionality of *polyAdapt* was illustrated using scenarios with different numbers of selection targets on a 1 Mb segment, which corresponds approximately to the size of a typical yeast chromosome, and evolution over 10 generations. The oligogenic simulations with 5 and 13 targets were included to illustrate the functionality of *polyAdapt*, while the polygenic case is a realistic architecture.

### Five Selection Targets

**Fig. 2.**
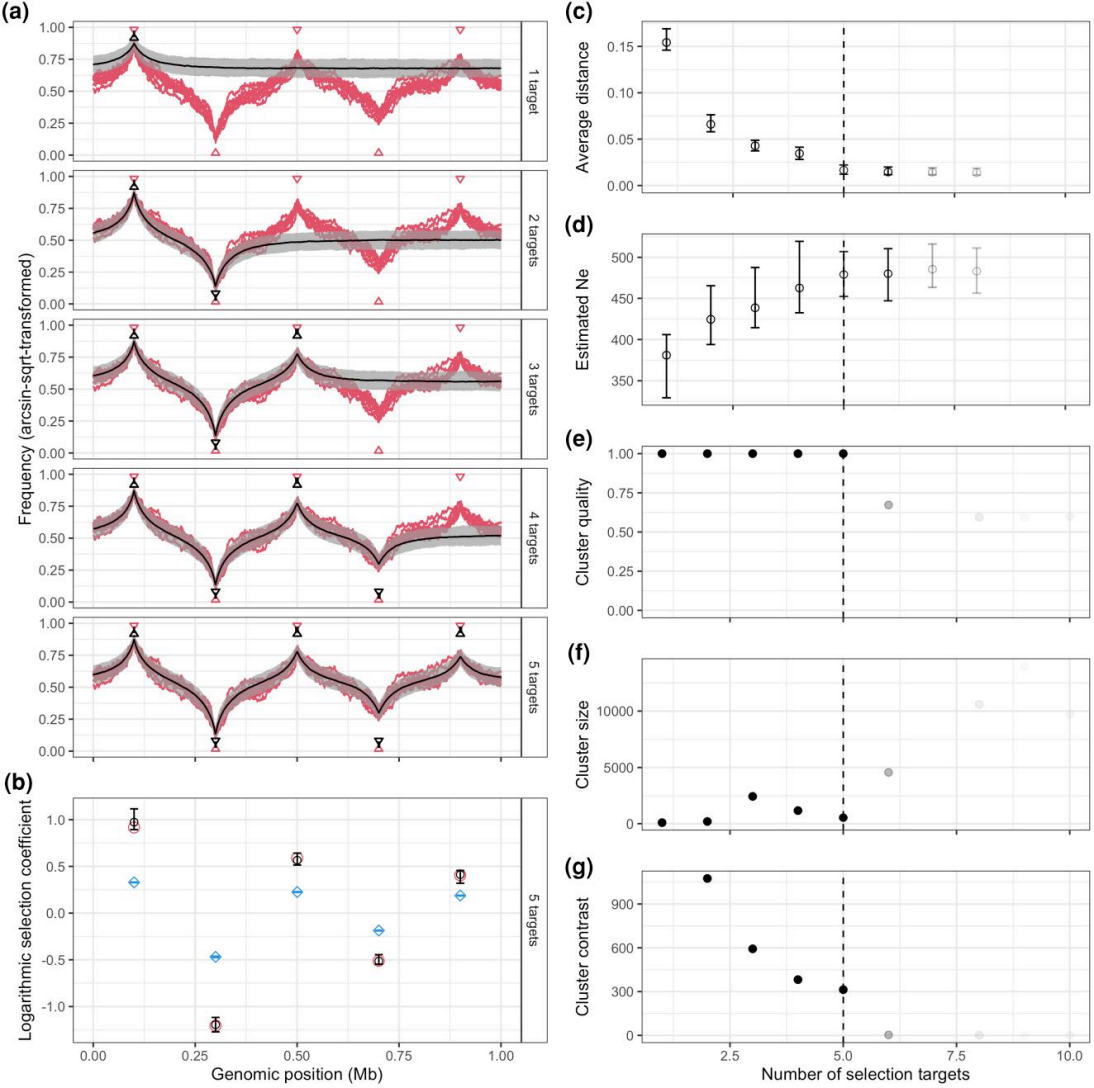
Functionality of *polyAdapt* with five selection targets. a) Each subpanel shows the frequency of 10 “empirical” replicates (red) after 10 sexual generation in a 1 Mb chromosome. The gray band shows the 95% confidence interval of 100 simulated replicates optimized for the assumed number of selection targets. Red triangles indicate the real selection targets and black triangles represent the clustered selection targets from 100 independent runs (each with 100 simulated replicates) of *polyAdapt* using the same empirical results. Horizontal error bars show the 95% confidence interval of the position across the 100 independent runs. Frequently the confidence intervals are too small to be seen on the plot. b) For each selection target, the real (red circles) estimated with *polyAdapt* (black circles) and estimated with PoolSeq (blue diamonds) targets with associated selection coefficients are shown. Vertical error bars indicate the 95% confidence interval of the inferred selection coefficient across 100 independent runs of *polyAdapt*. The criteria that can be used to infer the number of selection targets are show in c–g. The lower bound of selection targets can be inferred from the distance between empirical and simulated data c) or from the estimated effective population size d). The upper number of targets can be inferred from cluster quality e), cluster size f) and cluster contrast g).

Fig. 2 shows the allele frequencies of one of the two parental genotypes across a 1 Mb chromosome. The red lines show the frequencies of 10 replicate populations after 10 sexual generations. These data were generated by simulations and are intended to represent empirical data, therefore we refer to them as such. A 95% confidence interval of 100 simulated datasets is shown in gray. We deliberately used a larger number of simulated replicates than in the empirical data, as this reduces the influence of stochastic events in the simulations and consequently leads to more reliable inference. The subpanels of Fig. 2a show the best fit of the simulated data with different numbers of selection targets. Increasing the number of selection targets from 1 to 5 improves the fit between empirical and the simulated data, for both the selection targets and the non-selected regions separating them. The position of the inferred selection targets is remarkably precise and does not vary much between independent runs of *polyAdapt* when all five selection targets are specified (Fig. 2b). If fewer than five selection targets are assumed, the precision of some inferred selection targets is considerably lower. When the number of assumed selection targets exceeds five, the additional selection targets have very weak selection coefficients and were scattered across the entire chromosome (data not shown).

The importance of including linkage structure in this process becomes apparent when the selection coefficients inferred with *polyAdapt* are compared to the PoolSeq (Taus et al. 2017) estimates, which assume free recombination between selection targets. Only the *polyAdapt*-based estimates match the true values (Fig. 2b). The cause of this discrepancy is nicely illustrated in the panel with a single selection target. When simulating selection that matches the frequency change at the first selection target, the entire remaining chromosome is affected, not only the target of selection. Starting from a frequency of 50%, the frequency of the entire chromosome increases above 75% and the selection target can be recognized only by a peak with slightly larger frequency change than the rest of the chromosome. This linked selection effect explains why the alternation of positive and negative selection along the chromosome requires larger selection coefficients for all targets than under the assumption of free recombination (Fig. 2b). The selection coefficients estimated by *polyAdapt* are highly accurate (Fig. 2b).

Because *polyAdapt* provides estimates for a series of selection targets ranging from 1 to the user-defined upper bound, additional information is required to decide which number of targets explains the empirical data best. We propose five different criteria that could provide insights into this question.

The first criterion is the average distance between the empirical and simulated data. Unlike the estimation procedure for the selection coefficients, which minimizes the distance between the observed and simulated data only at the inferred selection targets, this measure uses all segregating SNPs. As expected, the distance decreases until the true number of selection targets have been reached. Adding more targets does not improve the fit (Fig. 2c). The effective population size (*N*_e_) estimated from the variance among replicates increases until the true number of loci has been reached and no further change is observed when a larger number of selection targets is assumed. Hence, both criteria are useful for estimating the lower bound of the number of selection targets.

The remaining three statistics require 100 independent runs of *polyAdapt* using the same empirical data. Reasoning that the similarity among the independent *polyAdapt* runs provides a measure of reliability, we cluster the positions of the inferred selection targets. Strong support is provided when all 100 runs place the selection targets at a similar position and this is reflected in a high cluster quality (Fig. 2e), small cluster size (Fig. 2f), and well-differentiated clusters, measured as cluster contrast (Fig. 2g). Since cluster-based criteria drop after the true number has been reached, they can be used to estimate the upper bound for the number of selection targets. All summary statistics identify five selection targets as the best solution.

### Thirteen Selection Targets

**Fig. 3.**
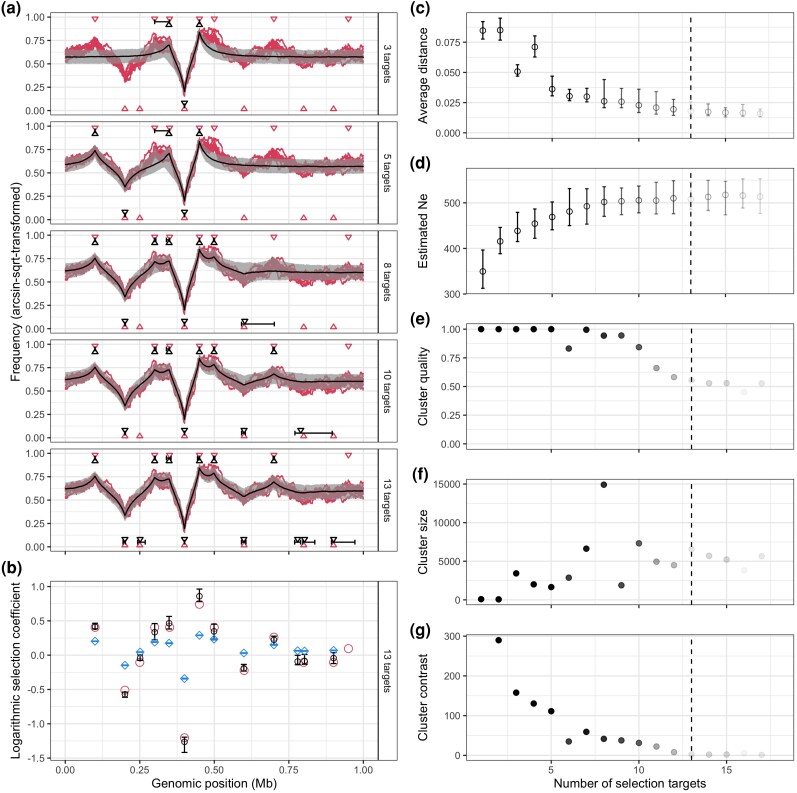
Functionality of *polyAdapt* with 13 selection targets evolved for 10 sexual generations. For detailed description of each panel, see Fig. 2.

To create a more challenging task, we used *polyAdapt* to characterize the adaptive architecture from simulations based on 13 selection targets. Like in the five-locus case, the overall fit of the estimated and true selection targets is remarkably good (Fig. 3). The 13-locus case has two interesting aspects that deserve more attention. First, some adjacent loci were selected in the same direction. Second, three weakly selected targets were adjacent to each other at the 3′ end of the chromosome. As expected, when only five selection targets were assumed, *polyAdapt* did not distinguish between adjacent selection targets with selection in the same direction. However, assuming more selection targets enabled the distinction between them. The three adjacent weak selection targets were not identified very reliably. One target was missed entirely, and the position of the other two was not very accurate (Fig. 3a). This suggests that 10 generations were not sufficient to resolve this genetic architecture, which is also evident from the summary statistics, which did not strongly support 13 selection targets.

**Fig. 4.**
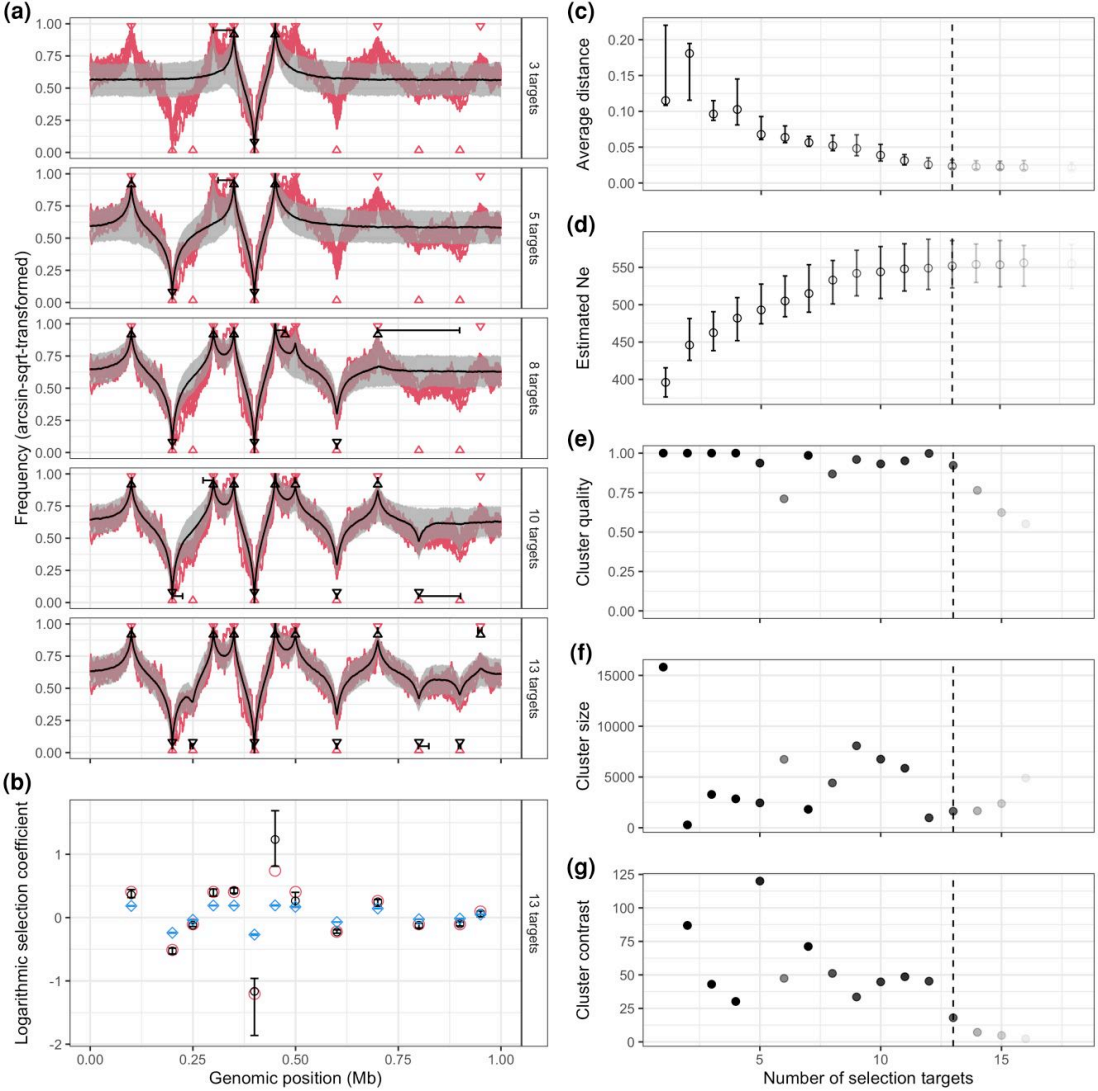
Functionality of *polyAdapt* with 13 selection targets evolved for 25 sexual generations. For detailed description of each panel, see Fig. 2.

Reasoning that a longer duration of the experiment might provide a better resolution, we extended it from 10 to 25 sexual generations (Fig. 4). In this data set, we made two interesting observations. First, the estimated selection coefficients of strongly selected loci were less precise than those from the 10-generation experiment, which we attribute to fixation in some of the replicates. Second, the weakly selected loci at the 3′ end of the chromosome were much more accurately characterized. After 25 sexual generations, the summary statistics provided a much cleaner support for the 13 selection targets than after 10 generations. In conclusion, *polyAdapt* performs very well even with more than a dozen selection targets, but longer experiments encompassing more sexual generations are required to provide a sufficiently strong signal.

### Fifty Selection Targets

**Fig. 5.**
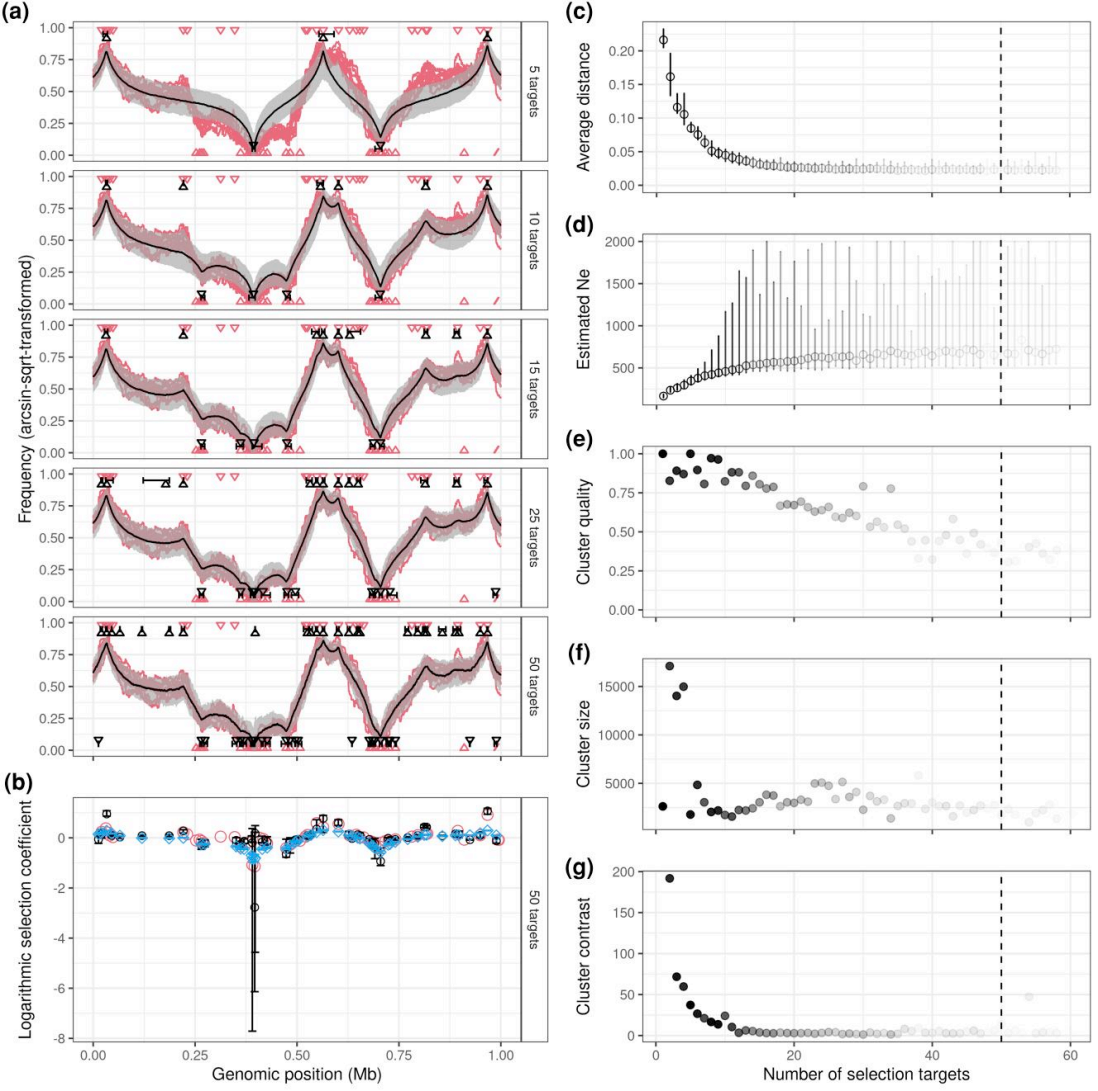
Functionality of *polyAdapt* with 50 selection targets (matching the allele frequency pattern of chromosome XII) evolved for 10 sexual generations. For detailed description of each panel, see Fig. 2.

In our third example, we explored a polygenic scenario with 50 selection targets (Fig. 5). While the scenarios with 5 and 13 targets were designed to illustrate the functionality of *polyAdapt*, this polygenic scenario aimed to match empirical results. Since the true number of selection targets is not known in real data, we used the allele frequency pattern of chromosome XII in the yeast data (Kosheleva and Desai 2018) as a template and placed 50 selection targets to match the empirical allele frequencies (see Materials and Methods for more details). With this configuration of selection targets and selection coefficients, we simulated 10 replicates to mimic empirical replicates, which were in turn used to evaluate the performance of *polyAdapt*. This strategy allowed us to use a realistic pattern of allele frequency change, but since the selection targets were known, the performance of *polyAdapt* could be evaluated.

As in analyses with fewer selection targets, we observed pronounced allele frequency changes across the entire 1 Mb chromosome, and the complexity of the frequency pattern was also similar to that seen in data sets with 5 or 13 targets. Therefore, the increased complexity of the genetic architecture does not result in a more complex selection response. This can be attributed to the clustering of loci with effects in the same direction (Fig. 5). Therefore, it is not surprising that the chromosome-wide allele frequency pattern can be faithfully reproduced with approximately 20 loci rather than 50. The good fit between empirical and simulated replicates with a smaller number of selection targets is also reflected in the average distance criterion, which plateaus after 20 loci. The effective population size and the cluster-based criteria are not very informative.

Since a small number of about 20 selection targets can accurately capture the chromosome-wide allele frequencies, adding more targets implies that the selection coefficient is divided among multiple targets. We evaluated this by comparing the average selection coefficient for different numbers of targets (Fig. S1). Consistent with our expectations, we notice that the average selection coefficient decreases with an increasing number of assumed selection targets. It is quite remarkable that additional selection targets are not randomly distributed over the chromosome, but form clusters. This is consistent with the idea that the selection response of a haplotype block can be explained either by a single, strongly selected target or by multiple weakly selected targets. We further noticed that, with many selection targets, the difference in selection coefficients estimated with or without considering linkage becomes negligible (Fig. 5b). We attribute this to the very small selection coefficients, which limit their influence on neighboring sites.

Increasing the duration of the experiments to 25 generations (Fig. S2) did not substantially improve the ability of *polyAdapt* to determine the number of contributing loci. Although some selection responses were more pronounced and the *N*_e_ criterion plateaued, the lower bound was estimated too low and no criterion provided information about the upper bound. We conclude that characterizing very complex adaptive architectures in full detail is difficult when the contributing loci are clustered. Resolving highly complex adaptive architectures would require larger population sizes in combination with more sexual generations. Nevertheless, it is quite remarkable that after 10 generations already about 20 distinct selected haplotype blocks can be distinguished.

### Empirical Yeast Data

We applied *polyAdapt* to experimental evolution data from yeast that was adapted for six sexual generations, focusing on chromosomes XIII and XVI, which exemplify two types of genotype frequency curves, namely one without and one with fixations of alleles from one genotype.

**Fig. 6.**
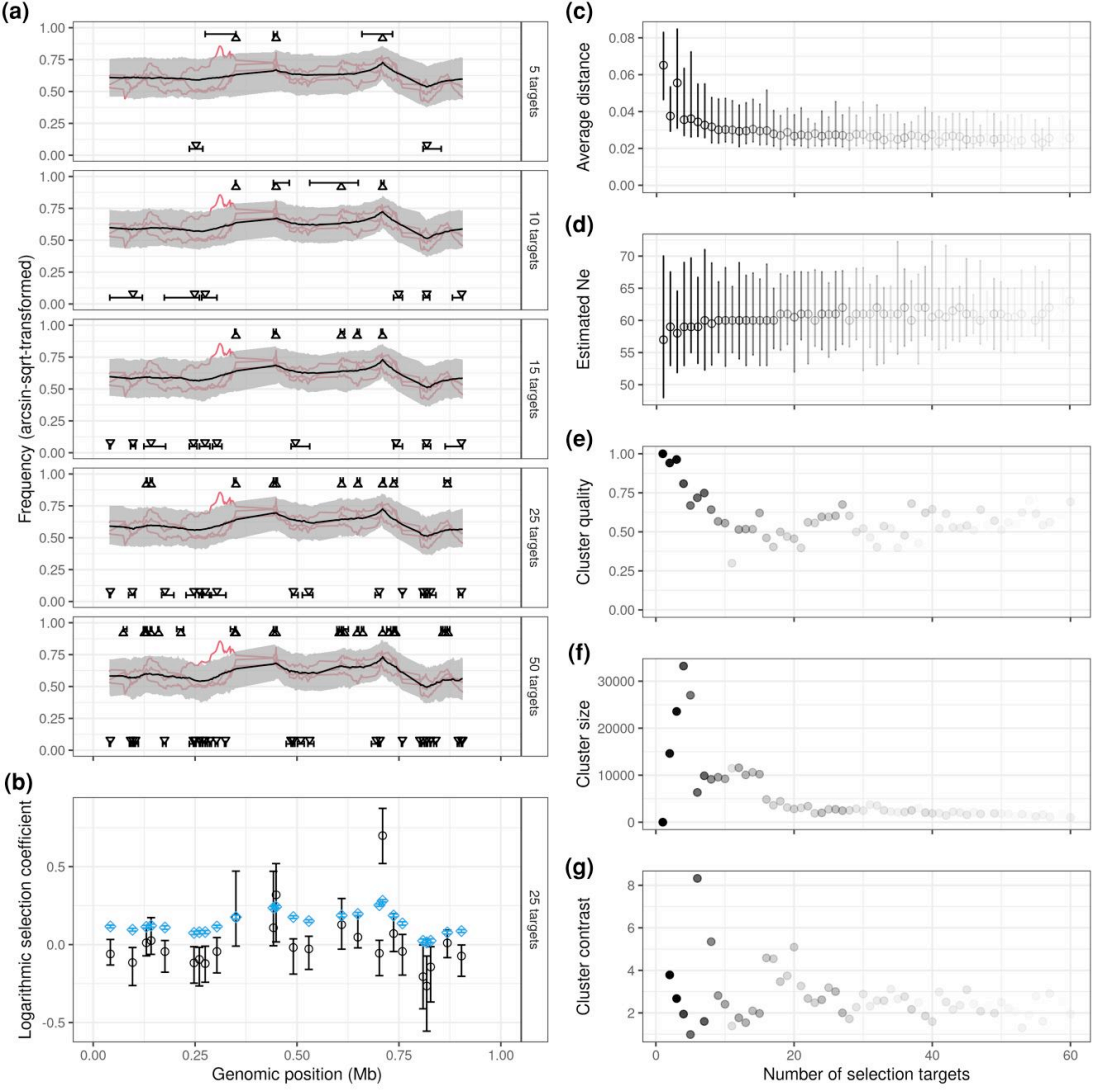
Inference of the adaptive architecture from yeast chromosome XIII with *polyAdapt*. After six sexual generations, the allele frequencies of three independently evolving replicates were determined by Pool-Seq. Allele frequencies of the W303 genotype were determined in windows as the mean of 250 SNPs. The analysis was performed in correspondence to the simulated data.

Chromosome XIII shows considerable heterogeneity in allele frequency among the empirical replicate populations (Fig. 6). These differences among replicates indicate substantial genetic drift, which is consistent with the unexpectedly small *N*_e_ estimate of about 60 individuals. A small effective population size implies more noise by genetic drift and fewer recombination events, both of which make the identification and characterization of selection targets challenging. Our analyses show that already with 20 selection targets, the average distance and *N*_e_ estimates plateau and do not change with additional selection targets. This indicates that a lower bound for the number of selection targets can be obtained. The cluster-based criteria, which could provide insight into the upper limit of the number of selection targets, were not informative in our analysis. This suggests that the empirical data do not provide sufficient information to make statements about the upper limit of selection targets.

For comparison, we also analyzed chromosome XVI (Fig. S3), which experienced larger allele frequency changes including some genomic positions which are fixed for one genotype. Analyzing data with fixations is more challenging, because there is more uncertainty about the position of the selection targets and their associated selection coefficients. This is reflected in our analysis, as neither the average distance nor *N*_e_ fully plateaus. This implies that adding more than 20 selection targets still improves the fit to the data. We caution, however, that the combination of a small population size and several fixations strongly limits the accuracy with which *polyAdapt* can estimate the underlying adaptive architecture. Interestingly, the estimated selection coefficients without accounting for linkage were almost consistently higher than those that accounted for linkage. This discrepancy, seen on both chromosomes, is due to a chromosome-wide increase in frequency of the W303 genotype, which was not observed in the simulations.

## Discussion

Identifying selection targets from genome-wide polymorphism data poses a multiple-testing challenge, as every polymorphic site is a potential candidate. Linkage further complicates the analysis by requiring joint consideration of linked loci. This complication is particularly pronounced in two-genotype experiments, where the confounding effects of linkage preclude the direct application of traditional molecular population genetics approaches to identifying selection targets and estimating their coefficients. Because exhaustively evaluating all possible combinations of selection targets and effects is computationally intractable, we developed an alternative approach that dramatically reduces the parameter space through targeted computer simulations.

Building on the fundamental population genetic principle that selection targets are expected to shift in frequency beyond neutral expectations, we identify selection targets iteratively and infer their coefficients by minimizing the distance between the allele frequencies of empirical replicates and simulations parameterized from the previous optimization cycle. This iterative strategy has a key advantage: it assesses the impact of linked selection before introducing additional targets in close proximity, substantially simplifying the estimation procedure.

For complex genetic architectures with few sexual generations, we observed that simpler architectures can produce trajectories indistinguishable from the true one. We attribute this limitation to two factors. First, when recombination events are scarce, linked loci remain insufficiently decoupled to distinguish the trajectory of a single strongly selected locus from that of multiple linked loci with individually small effects. Longer experiments, larger population sizes, and the resulting increase in recombination events can improve both resolution and accuracy; larger populations additionally reduce the influence of genetic drift. Second, adding a single small-effect locus yields only marginal improvement in the fit between empirical and simulated data, making haplotype blocks with large net effects readily detectable while those with small net effects remain elusive. Additional replicates and generations could improve performance in such cases.

Importantly, the uncertainty arising from complex genetic architectures reflects the intrinsic difficulty of the problem itself, not any deficiency of *polyAdapt*.

Although two-genotype experimental evolution data are still relatively uncommon, we anticipate that the clean, parallel selection signatures make this approach an attractive design for experimental evolution studies. We also anticipate, however, that future experiments will also not be sufficiently powerful to distinguish individual selection targets of a polygenic trait. This implies that *polyAdapt* will estimate the joint effect of all contributing loci on a haplotype block. This has two important consequences. First, while the specific positions of selection targets cannot be treated as definitive, since any variant in a haplotype block may be the causal allele, their inferred locations are surprisingly reproducible across independent *polyAdapt* runs. This consistency holds for a set number of targets, though it does not achieve base-pair resolution (Figs. 4 and 5). Second, only a lower bound can be obtained for the number of loci contributing to the selected trait(s). Hence, we caution that the estimated number of selection targets should not be interpreted as an exact count. First, the number of selected loci may be underestimated if multiple targets reside within the same haplotype block. Second, the proposed metrics for the upper and lower bounds of target counts are strictly qualitative. Nevertheless, we do not view this as a major limitation, as inferring the precise number of selected loci for polygenic traits is notoriously challenging and heavily dependent on the threshold used to define small-effect variants. Instead, two-genotype experiments combined with *polyAdapt* are well-suited to characterize the adaptive genomic landscape at a remarkable, albeit incomplete, resolution.

The analysis of the empirical yeast data suggests a highly polygenic architecture, with a lower bound of 20 targets per chromosome. Although Kosheleva and Desai (2018) employed a different approach estimating the number of selection targets, their estimates of 25 to 100 selection targets per megabase are remarkably consistent with ours. We were surprised by the small *N*_e_ estimates derived from the yeast experiments. While our model did not explicitly account for the asexual phase of the experiment, we consider it unlikely that this significantly contributed to the small *N*_e_ estimates, as population sizes remained large during asexual growth. The drastic reduction in population size occurs primarily during the sexual generation. A more plausible explanation is that selection on other chromosomes reduced genome-wide *N*_e_ which is also reflected in the heterogeneity among replicates of the focal chromosome. We validated this hypothesis through simulations where selection operated on multiple chromosomes, while *N*_e_ was inferred from a single chromosome. Although the *N*_e_ estimate was likely biased by selection on other chromosomes, our simulations demonstrated that the selection coefficient estimates remained robust and unaffected by this unaccounted selection (data not shown).

Our inference of the adaptive architecture relied solely on allele frequency shifts in evolved replicates after 10 or 25 generations, starting from a common founder population. We anticipate that incorporating intermediate time points would yield deeper biological insights, particularly by enabling the inference of temporal dynamics in selection coefficients. Such dynamics are critical for elucidating the adaptive architecture: dominance and epistatic interactions predict frequency-dependent selection responses, which manifest as shifts in selection coefficients over time. Furthermore, selection coefficients are expected to evolve following a shift in the trait optimum. Immediately after the shift, selection is strongest and approximates a selective sweep scenario (Jain and Stephan 2017). However, as the population approaches the new trait optimum, the selection pressure on individual loci decreases (Franssen et al. 2017b; Hayward and Sella 2022).

The current version of *polyAdapt* assumes a constant selection coefficient across generations, but future versions will incorporate the analysis of time series data, providing the opportunity to uncover changes in selection coefficients across generations. Additionally, it will be possible to incorporate explicit models of dominance, and the expectations for polygenic adaptation following a shift in trait optimum. More broadly, the flexibility of the SLiM simulation framework enables the testing of a wide range of hypotheses regarding the underlying adaptive architecture using two-genotype experimental evolution data. In short, *polyAdapt* represents a powerful approach for characterizing the adaptive architecture in an experimental evolution framework, where adaptation is driven by multiple loci.

## Materials and Methods

Two-genotype experiments start by mating individuals that are homozygous for one of two distinct genotypes (e.g., A/A or T/T). Hence, a list of marker SNPs representative for one of the genotypes can be generated. While in the first generation, all genotype-specific SNPs are in full linkage disequilibrium, in subsequent generations, recombination uncouples genotype-specific SNPs. Genome-wide allele frequency estimates can be obtained by sequencing pools of individuals with short-read sequencing technologies (Pool-Seq) (Schlötterer et al. 2014, 2015). Because neighboring SNPs remain highly correlated, it is possible to increase the accuracy of allele frequency estimates by averaging across multiple neighboring SNPs, replacing single marker SNPs with marker SNP windows. The empirical data can consist of a single replicate or multiple replicate populations, which started from the same founder population and evolved for the same number of generations in the same environment. The inference of *polyAdapt* relies on the contrast of the ancestral population to data from a single evolved generation (intermediate generations are not considered), either with a single or multiple replicates.

To identify those marker SNP windows that carry putative selection targets, we developed *polyAdapt,* an *R* script (supplemental information; available from https://github.com/orgs/popgenvienna/repositories, where future updates will be also published), which implements the procedure described in detail below.

### The *polyAdapt* Algorithm: Iteratively Enlarge a Set of Selection Targets

Given a set of SNPs distributed over a genome and corresponding empirical genotype frequencies at a certain experimental generation (possibly from several replicates), we build a set of selection targets though an iterative procedure that adds a new target position in each round.

The first target is placed at the position with the largest frequency difference between simulated (under neutrality) and empirical, evolved replicate(s). An optimized selection coefficient is obtained by maximizing the fit between the empirical SNP frequencies and 100 simulated replicates at the target position using the Nelder–Mead algorithm (Nelder and Mead 1965; Nash 1979), which is implemented in the *R* function stats::optim(). The quality of fit is determined by the distance between empirical and simulated data (see below for the calculation of the distance). Thus, during the optimization, the distance is evaluated repeatedly using new simulations based on improved estimates of the selection coefficient. The number of optimization rounds is determined by the optimizer.

In the case of a single empirical replicate, the distance *d*_M_ is the absolute frequency difference between the median of the 100 simulated replicates and the empirical data at the target position. The distance *d*_M_ is used by the optimizer to find the selection coefficient that best matches simulated and empirical data.

In the case of multiple empirical replicates, we use $d_{c}=1/(1/d_{M}+1/d_{\rho })$, where *d*_M_ is estimated from the median frequencies of the empirical replicates and the median frequencies of the simulated replicates at the target positions, and where $d_{\rho }=-\log\;\rho$ with $\rho =U/U_{\max}$, the normalized rank-sum-based Mann–Whitney *U* statistic that measures the overlap between simulated and empirical replicates at the target positions (Mann and Whitney 1947). The equation of *d*_c_ is designed to capture two aspects of the data. First, it accounts for differences in allele frequency at the selection targets seen across all replicates. Second, it accounts for the (dis)similarity among replicates at the selection targets, thus preventing the optimizer from overfitting the median.

Simulations are conducted with SLiM v4 (Haller and Messer 2023) based on a user-defined recombination rate. The slim script provided by *polyAdapt* (mix2pops.slim) uses the standard Wright-Fisher model with a panmictic population, 50:50 sex ratio, additive allele effects (*h* = 0.5) and no epistasis and no new mutations. Autosomes and “X” type sex chromosomes are both supported, although only the former was used in this study. This SLiM script can easily be replaced by a user-defined script to implement more complex scenarios.

The effective population size (*N*_e_) is a key factor determining the similarity of allele frequency changes in replicate populations. Therefore, *polyAdapt* estimates *N*_e_ when multiple empirical replicates are available. Using all segregating sites, selected and neutral ones, the variance in allele frequency among replicates is used to estimate *N*_e_. Based on the difference in mean variance in the empirical (*f*_e_) and 10 simulated (*f*_s_) frequencies across all segregating sites, the optimizer infers *N*_e_ by minimizing $\log^{2}\;\bar{\mathrm{var}(f_{s})}/\bar{\mathrm{var}(\;f_{e})}$. Ten replicate simulations are sufficient for reliable *N*_e_ estimates, because all segregating sites are used (We note that this procedure estimates *N*_e_ in the presence of selection, rather than under neutrality.). After resetting *N*_e_ to the new estimate and outputting the position of the selection target together with the optimized selection coefficient and the optimized *N*_e_, the first round is complete.

A new round is started by identifying a new selection target based on the maximum allele frequency difference between the observed data and 10 replicates simulated with the parameters optimized in the previous round. First, the selection coefficient is estimated for the new target only, while keeping the estimated selection coefficients for the other selection targets from the previous round. Then, the selection coefficients are optimized for all selection targets by minimizing the average distance (*d*_M_ or *d*_c_). This strategy reduces the evaluated parameter space, making the optimization with multiple selection targets computationally feasible. The *N*_e_ optimization concludes the round as described above. New selection targets are identified and subsequently optimized until the user-defined maximum number of selection targets has been reached. The optimum number of selection targets is then determined using several criteria described below.

### Evaluation of *polyAdapt* With Known Selection Targets

We evaluated the performance of *polyAdapt* with known selection targets and their associated selection coefficients. We generated synthetic data sets with 5 and 13 selection targets by placing them manually on a 1 Mb chromosome. Ten replicates, each with 250 individuals from each of the two diploid homozygous genotypes, were evolving for 10 generations (using SLiMv4, *loc. cit.*; slim script mix2pops.slim in SI). We use a constant recombination rate of 5 × 10^−6^/bp approximating the recombination rate in yeast (Jinks-Robertson and Petes 2021). The selection coefficients in all simulations were manually chosen. The five-target set has alternating strong targets resulting in clear frequency peaks and valleys, while the 13-target set also contains neighboring loci with effects in the same direction and very weakly selected loci. These two data sets were mainly used to illustrate the functionality of *polyAdapt* without the complication of clustered selection targets.

For more biological realism, the 50-target set was modeled based on the allele frequencies of chromosome XII in the empirical yeast data set (Kosheleva and Desai 2018). We used the empirical data to estimate the position and selection intensity of 50 loci with *polyAdapt*. After some minor adjustments, the estimated positions and selection coefficients were used to simulate 10 replicates, which were treated as empirical data in the evaluation of *polyAdapt*. Since the position and selection strength were known, we were able to evaluate the performance of *polyAdapt*.

### Criteria for Estimating the Number of Selection Targets

#### Average distance

The first criterion is the average distance between empirical replicates and simulations across all segregating sites, the same measure as used to optimize selection coefficients (where the average is taken only across target sites). The average distance decreases as selection targets are added, eventually reaching a plateau. Therefore, the average distance provides insights into the minimum number of targets.

#### Effective population size

The second criterion is the effective population size estimated at the end of each round. Replicates exhibit higher variance under neutral evolution than under selection, because selection pushes allele frequencies at target locations toward certain values across replicates, and so constrains the possible realizations and thus the inter-replicate variance. Therefore, *N*_e_ is generally underestimated as long as selection remains underestimated and reaches a plateau once enough selection targets are included in the simulations. Like the average distance above, *N*_e_ thus provides insights into the minimum number of targets.

Neither of the above criteria provides information about the upper bound of the number of selection targets. This is because additional targets can always be added with near-zero selection coefficients without changing the average distance or the *N*_e_ estimate much. Insight into the upper bound must come from repeated runs of *polyAdapt*, which are expected to agree only for high-confidence targets.

To integrate information from repeated runs, we aggregate all target positions for target sets of size *k* from *n* = 100 repeated runs, each with 100 simulated and the same empirical replicates, into *k* clusters using the Clara_Medoids() function from the *R* package *ClusterR* v1.3.4. We reason that the properties of the target clusters provide the criteria to estimate the upper bound for the number of selection targets because surplus targets will be placed more randomly than targets that actually shaped the selection response. The criteria are described below.

#### Cluster quality

The fraction of *polyAdapt* runs placing a selection target in the chromosomal region as defined by the cluster. More reliable targets are expected to have a higher cluster quality. Importantly, when the number of selection targets is underestimated, the cluster quality is usually high, but when too many targets are assumed, cluster quality is decreasing. Thus, cluster quality is informative about the upper bound for the number of selection targets.

#### Cluster size

Cluster size is the average within-cluster distance. A small cluster size implies that independent *polyAdapt* runs place the targets at very similar positions. Like for cluster quality, this criterion is particularly helpful for defining an upper bound of the number of selection targets.

#### Cluster contrast

Cluster contrast is the average between-cluster distance (to the nearest neighbor) divided by the cluster size. Tighter and better-separated clusters result in a higher cluster contrast, which is most informative for defining the upper bound for the number of selection targets.

### Evaluation of the Fit of the Inferred Selection Targets

The target clusters obtained to calculate the criteria for the upper bound of target numbers can be also use to evaluate the accuracy of the position of the selection targets and the associated selection coefficients. Each cluster contains the inferred selection targets from 100 independent *polyAdapt* runs, each with 100 replicates, on the same empirical data. The fit of the inferred selection targets can be expressed either by the mean and variance of the target positions or the mean and variance of the associated selection coefficients. We use these means and variances to assess the accuracy of *polyAdapt* reasoning that accurate inferences are associated with smaller variances among the 100 independent *polyAdapt* runs.

### Application to Experimental Data

We applied *polyAdapt* to a publicly available data from a two-genotype experiment with diploid brewer's yeast (Kosheleva and Desai 2018) obtained from the NCBI (PRJNA359887). We used the “frequent sex” subset, which comprises short read data sets for the ancestral population (d1, sample 536) and three replicates evolved for 240 generations (samples 504, 508, 512). Since only one of the two founder genotypes was available (sample 537), we used a W303-K6001 assembly (PRJNA83445) (Ralser et al. 2012) as the second genotype, which differs only slightly from the W303 strain used in the experiment (Desai, *personal communication*).

Marker SNPs for the founder strains were determined by aligning the two founder genotypes to the *S. cerevisiae* reference R64 (NCBI accession number GCF_000146045.2) also including the 2-micron circle plasmid A364A (NC_001398) and a few specific virus genomes (NC_003745, NC001782, NC_004050, NC_001641). Biallelic marker SNPs were extracted from the aligned reads by conditioning on a minimum read count of 5 and a minimum difference of 80% in allele frequency. The frequencies of the marker SNPs for the genotype W303 were estimated from the aligned Pool-Seq reads from generation 240.

The short read data sets were processed in a standard pipeline, which includes trimming with in-house scripts (with the modified Mott algorithm (Kofler et al. 2011; Gomez-Sanchez and Schlötterer 2018) to a minimum base quality of 13, TruSeq-3 adapters, minimum read length 35), mapping to the reference with bwa-mem2 v2.2.1 (Vasimuddin et al. 2019) (arguments: -M -L10,10), and duplicate removal with samtools-markdup v1.15.1 (Li 2018) (arguments: -S --mode s --include-fails).

The W303 assembly was mapped with minimap2 v2.17-r941 (Li 2018, 2021) (arguments: -ax asm5) to the same reference R64.

Pileup files were generated from all mapped files using bcftools-mpileup v1.15.1 (arguments: --skip-all-unset 0x2 -q 10 -Q 20 -D -a DP,AD,QS,SCR). The pileup files were merged and post-processed with an in-house script (supplemental information), which removes the placeholder allele “*”, all monomorphic positions, and calculates the allele frequencies. Then, we further filtered them with bcftools-filter v1.15.1 (Danecek et al. 2021) to extract all biallelic SNPs not close to indels or in masked regions of the reference (arguments: ‐‐snpGap 20 -T not_masked.bed.gz -i "N_ALT = 1 & REF ! = 'N' & TYPE = 'snp'").

From the merged biallelic SNP pileup, frequencies of the W303 genotype were estimated from non-overlapping 250-SNP windows using the script *haploFreq.R*, which implements a refined version of the estimation method introduced in (Mazzucco et al. 2020).

The meiotic recombination rate of *S. cerevisiae* is 7 × 10^−6^ based on crossover estimates in Mancera et al. (2008). Mitotic recombination rate is lower by orders of magnitude and can be neglected in comparison (Jinks-Robertson and Petes 2021). The “frequent sex” sub-experiment has 40 asexual generation followed by one sexual generation. Therefore, generation 240 corresponds to six sexual generations only (Kosheleva and Desai 2018). Therefore, we model the evolution of this “frequent sex” scenario as normal diploid evolution for six generations using the meiotic recombination rate.

### Computational Costs

All estimations with *polyAdapt* were run on a cluster of servers with 64-core Epyc 9 processors. A single *polyAdapt* run using 10 cores took less than 6 h to identify 50 targets in the yeast data and about 15 h to identify 100 targets. In comparison, for the 50-target scenario with its nearly 10-fold higher *N*_e_ estimate, identifying 50 targets took about 10 h.

## Supplementary Material

evag132_Supplementary_Data

## Data Availability

No new data were used in this publication.
