# Supplementary material for: PolyAdapt: Characterizing Polygenic Adaptive Architectures in the Presence of Strong Linkage Disequilibrium
Source: Genome Biol Evol. 2026 Jun 9;18(6):evag132. doi: 10.1093/gbe/evag132 (PMC13281831; doi:10.1093/gbe/evag132)
Supplement: evag132_Supplementary_Data [file evag132_supplementary_data.zip › supplementary figures.pdf]

Supplementary figures

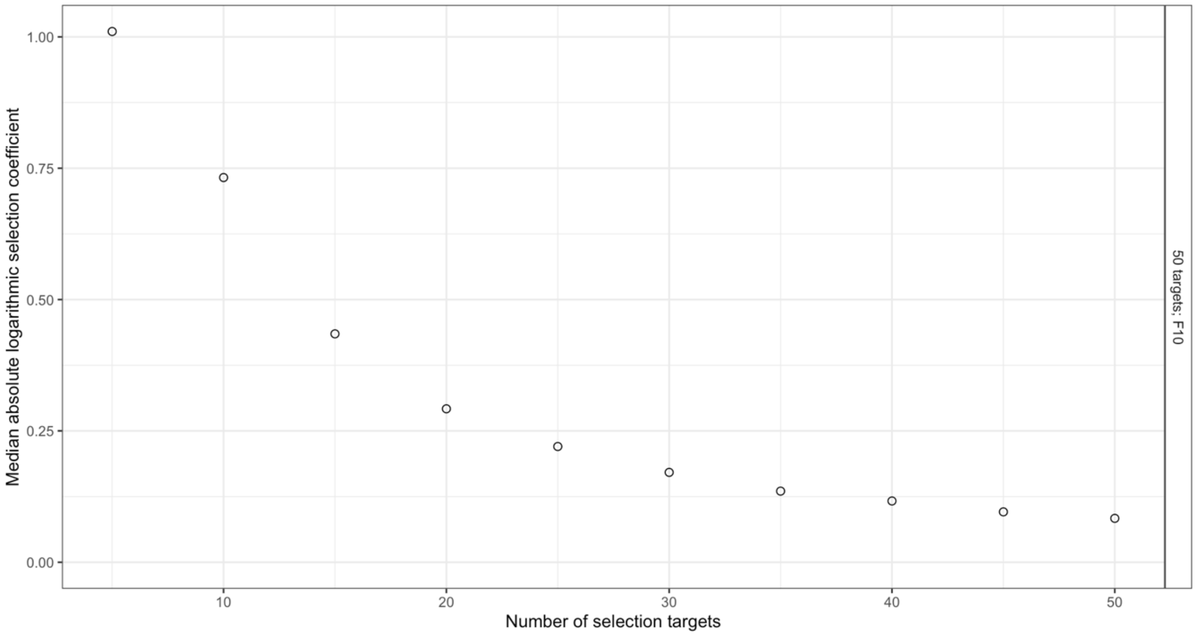

Figure S1: Median selection coefficient decreases with an increasing number of assumed selection targets. *polyAdapt* was applied to 10 replicates simulated with 50 selection targets.

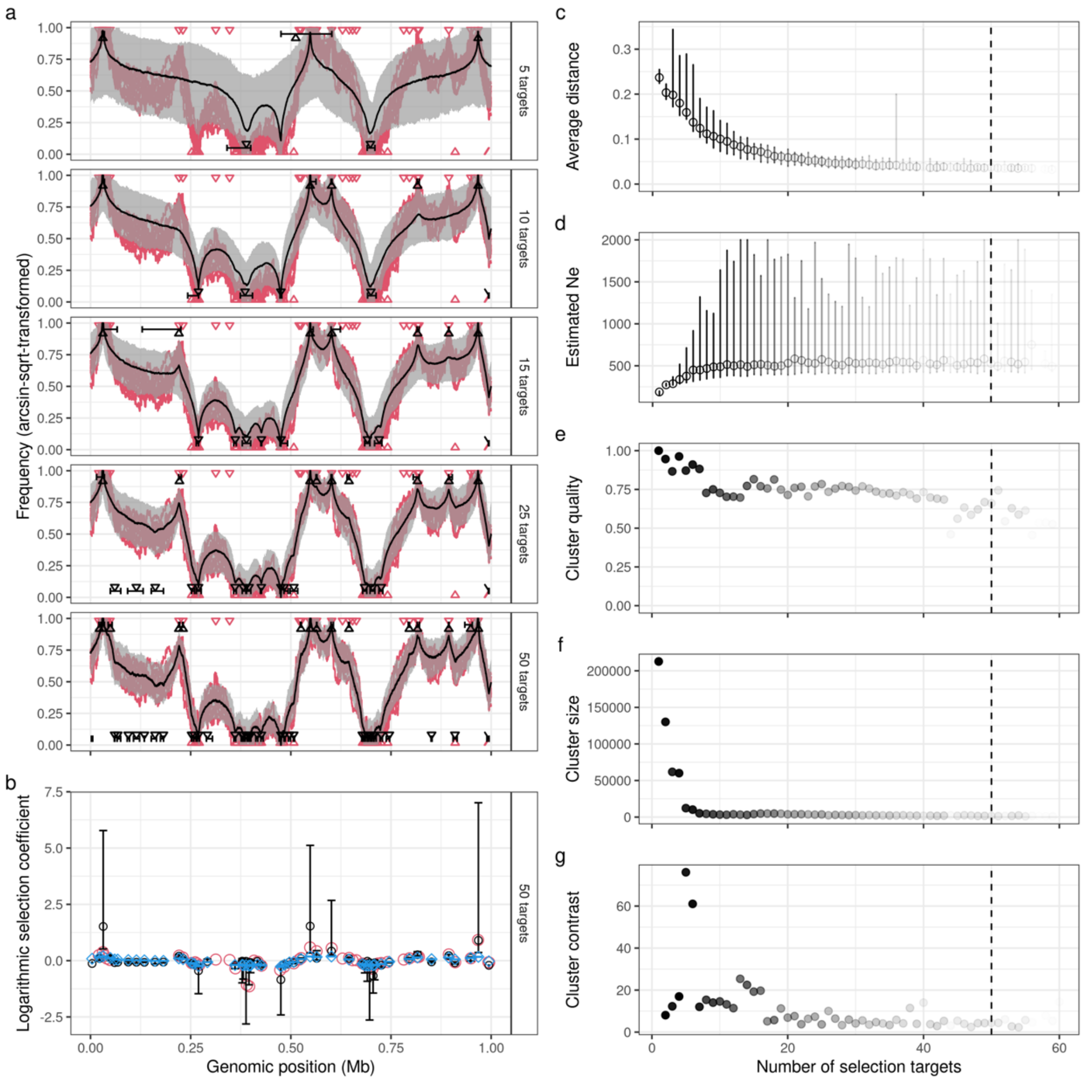

Figure S2: 50 selection targets evolved for 25 generations.

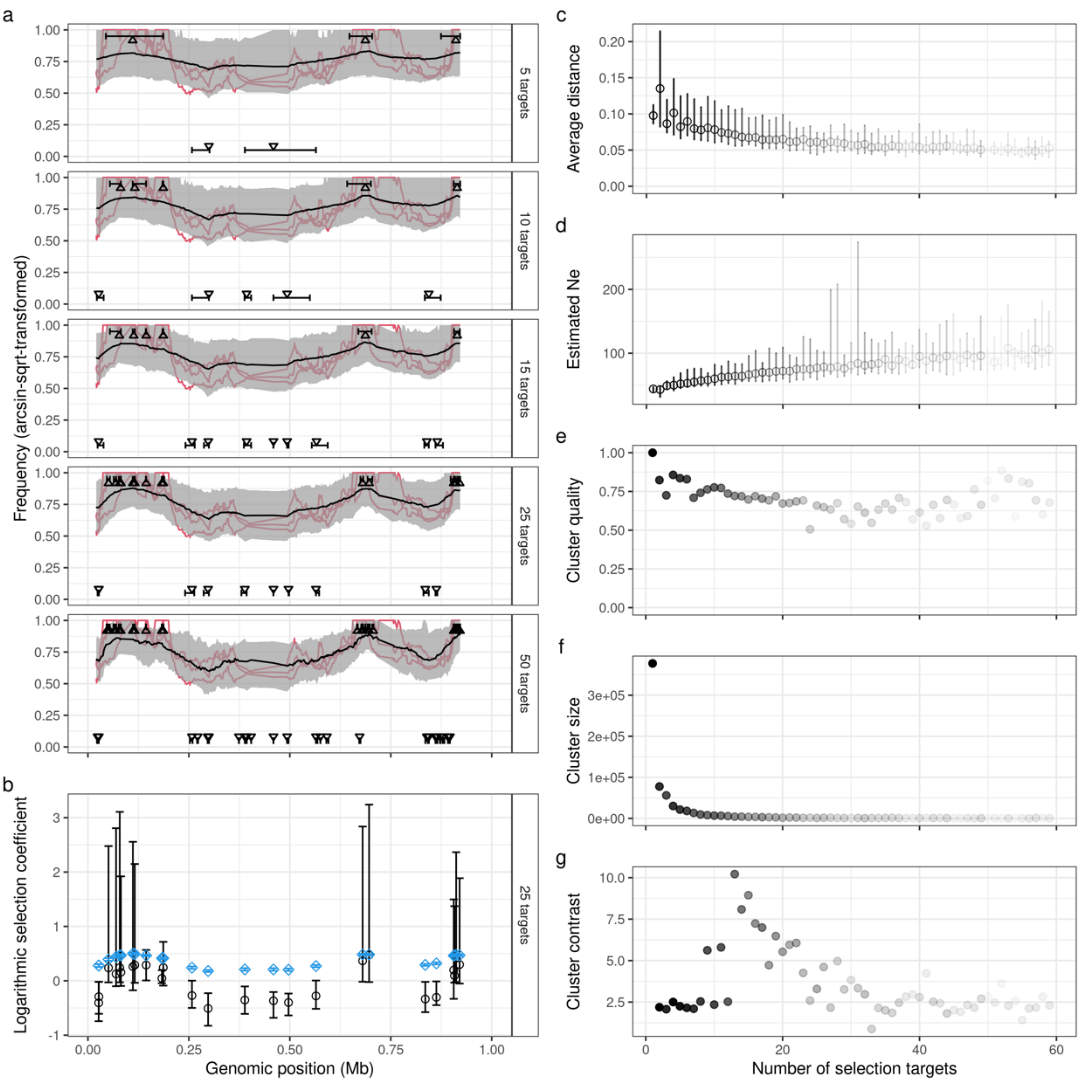

Figure S3: Yeast chromosome XVI

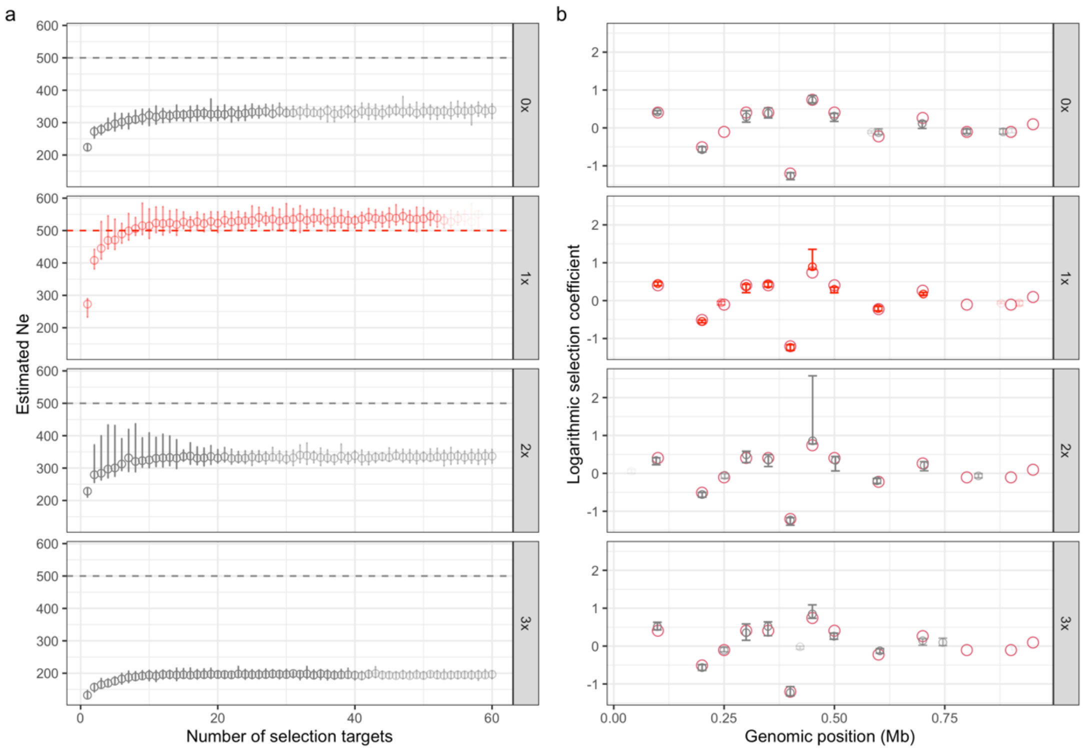

Figure S4: The effect of ignoring other chromosomes on estimating N_e and selection targets. Estimation was done as for main Figs. 3d and 3b, to which the panels labelled "1x" and highlighted in red also directly correspond. For the other three rows, the data on which the estimation was based had been obtained with a background of additional, unobserved, chromosomes, specifically: a second copy of the observed chromosome with exactly inverted selection targets, so that the total fitness was exactly equal to 0 ("0x"); a second and third copy of the observed chromosome, so that the total fitness was doubled and tripled, respectively ("2x" and "3x"). The effect on N_e estimation is undeniably strong (a), but there is essentially no effect on the estimated selection targets (b).
